# Effect of Balloon-Blowing Exercise on Oxygen Saturation in COVID-19 Patients

**DOI:** 10.7759/cureus.40250

**Published:** 2023-06-11

**Authors:** Anand Misra, Rajni Pawar, Akshay Pal

**Affiliations:** 1 Department of Physiotherapy, Sri Aurobindo Institute of Allied Health and Paramedical Sciences, Sri Aurobindo University, Indore, IND

**Keywords:** spo2 saturation of oxygen in blood, balloon exercises, covid-19, sars-cov-2, coronavirus

## Abstract

Background

Balloon therapy is an empirical example that patients with respiratory tract diseases can use to improve their daily care. The diaphragm and ribs are expanded and raised by the intercostal muscles, which are activated during the balloon-blowing exercise. This makes it possible for the lungs to inhale oxygen and exhale carbon dioxide. Therefore, this study aimed to investigate the impact of balloon-blowing exercise on blood oxygen saturation in stable, non-intubated COVID-19 patients.

Methodology

Considering the guidelines approved by the Ministry of Health and the Government of India, 250 COVID-19 patients with an age group between 20 and 80 years based on the inclusion criteria were taken. After the collection of baseline data consisting of oxygen saturation (SpO2) measured with a portable pulse oximeter, the balloon-blowing exercise with conventional physiotherapy was given for 30 minutes, and clinical data of SpO2 were re-collected.

Results

The pre- and post-results of balloon blowing exercise, along with conventional physiotherapy in 250 patients, were assessed by measuring SpO2. The results demonstrated that the balloon exercise was found to be effective, as oxygen saturation improved after the application of the balloon exercise. Analysis revealed that pre- and post-values involving 95.685±1.645 and 98.123±1.445, respectively, differed significantly. The results demonstrated that the application of balloon exercise to COVID-19 patients led to a considerable improvement in SpO2.

Conclusion

The difference between the pre- and post-values of SpO2 was found to be significant, which suggests that balloon exercise is a low-cost physiotherapy strategy that can be utilized to enhance oxygen saturation in COVID-19 patients. This demonstrates that the need for ICU treatment can be lessened, and consequently, the burden on healthcare facilities can be reduced.

## Introduction

On March 11, 2020, the World Health Organization Emergency Committee declared the severe acute respiratory syndrome coronavirus 2 (SARS-CoV-2) coronavirus disease 2019 (COVID-19) outbreak a pandemic, which had its origins in the People's Republic of China's Hubei Province [1,2]. In contrast to other respiratory viruses, it spreads from person to person and occurs 2 to 10 days before the person starts to experience symptoms [3]. Through the eyes, hands, nose, and mouth, the virus is transferred from one person to another. Patients with COVID-19 might have respiratory infections and influenza-like diseases in addition to fever (89%), wet and dry cough (68%), shortness of breath (19%), sputum production (34%), and fatigue (38%) [4]. The severity of the sickness might range from an infection with no symptoms or a minor upper respiratory infection to severe viral pneumonia with respiratory failure and/or death. Recent studies suggest that 5% are thought to be critical, requiring ventilation and life support, 15% are thought to be severe (infections requiring oxygen), and 80% of cases are asymptomatic or mild [3]. Pneumonia is the most frequent complication in patients with severe COVID-19, but additional issues can include sepsis, acute respiratory distress syndrome, and septic shock, as well as acute kidney injury, cardiac injury, and multiple organ failure [5,6].

Various lung therapies have emerged to treat these complications, and among them, respiratory physiotherapeutic interventions consisting of muscle-strengthening exercises have been demonstrated to provide several therapeutic benefits. Improved exercise performance, pulmonary function, and respiratory muscle strength, respiratory function are a few of these outcomes, along with maintenance or improvement of oxygen saturation and correction of aberrant breathing patterns [7]. Regular exercise strengthens muscles and maintains a healthy heart and lungs [8]. As a result, physical fitness increases, and the body gets better at transferring oxygen to working muscles through the bloodstream. Patients with COVID-19 benefit significantly from physiotherapy, and frequent breathing exercises may help them recover more quickly. Blowing balloons is one of the simplest breathing exercises, and it can be done by everyone, from young children to the elderly [9]. 

Exercises involving inflating balloons strengthen the intercostal muscles, which spread and elevate the ribcage and diaphragm. This makes it possible for the lungs to inhale oxygen and exhale carbon dioxide [10]. These individuals, who frequently have low blood oxygen due to breathing issues, may benefit from an early breathing exercise session to enhance oxygen circulation [11]. By encouraging balloon-blowing activity, an economical alternative to breathing exercises, the odds of developing a major illness may be reduced by strengthening the body's respiratory muscles during COVID-19. As a result, the study's goal was to assess the effect of balloon-blowing exercise on COVID-19 patients to increase oxygen saturation levels.

Exercises involving blowing balloons increase the body's oxygenation, allowing the patient to exercise for extended periods without getting out of breath or exhausted. By dissolving glucose and generating fuel for muscles, oxygen restores energy to cells and muscles. Muscles are going to provide themselves with additional energy stores when oxygen is abundant, which boosts lung endurance. By committing to a regular habit of inflating 10 or 20 balloons, lung capacity can be gradually expanded, as well as the ability to sustain an adequate quantity of oxygen over time [11]. By performing balloon-blowing exercises, respiratory muscles can be improved, which contributes to lifting the ribcage so that the lungs can expand. All the muscles are effectively worked out as the balloons slowly blow up, increasing stamina, lung capacity, and oxygen saturation [11].

## Materials and methods

After approval from the Institutional Ethical Committee (IEC) with reference number (/SAIMS/IEC/2020/471), an experimental/single group pre-and post-test study was carried out at the allocated COVID-19 hospital at Sri Aurobindo Hospital, Indore district, for a duration of three months, that is, from July 2020 to September 2020. Overall, 250 COVID-19 patients were involved in the study by non-random convenient sampling. The inclusion criteria considered were both male and female known COVID-19 patients aged between 20 and 80 years, spontaneously breathing, COVID-19 positive in RT-PCR, and diagnosis based on clinical symptoms or a CT scan or high-resolution computed tomography HRCT chest. Critically ill COVID-19 patients on bi-pep or a ventilator, as well as patients who were intubated and uncooperative, were excluded from the study. The materials used involved a pulse oximeter (Accu Sure) and balloons. The total duration of treatment was 30 minutes, consisting of 10 repetitions in one set and the rest of 5 min in one set of training, for a total of three sets of training.

After wearing personal protective equipment and following proper hygiene procedures, the oxygen saturation levels of COVID-19 patients were measured using pulse oximeters. Along with conventional respiratory physiotherapy, specific breathing exercises consisting of the balloon-blowing exercise were demonstrated and explained. All patients were given individual balloons to perform breathing exercises, taken to a sitting position and asked to perform balloon-blowing exercises along with conventional physiotherapy. Oxygen saturation, heart rate, and respiration were monitored during the procedure. For the balloon blowing exercise, patients were asked to sit comfortably, and the balloon was given to them individually. The patients were instructed to place the balloon in their mouths, inhale deeply through their noses, and place their tongues on the roof of their mouths. Maximum volume intake for three to four seconds was followed by an exhale for 8 to 10 seconds, with an interval of two to three seconds at the conclusion of the exhalation. With adequate rest of more than a minute in between sets, three sets of 10 repetitions each were completed. A pulse oximeter was used to monitor oxygen saturation, which was an outcome measure following the balloon-blowing exercise.

The conventional respiratory physiotherapy exercise regimen consisted of thoracic expansion exercises, diaphragmatic breathing, active cycle of breathing techniques, active range of motion of upper and lower limbs, forced expiratory technique, and glossopharyngeal breathing with a frequency of one set of 5-10 repetitions and was performed two times a day. After exercise, oxygen saturation was again measured by a pulse oximeter. The maneuver and the clinical monitoring of the patient's tolerance to the balloon-blowing exercise, along with conventional respiratory physiotherapy, were done under supervision, including monitoring of saturation during and after the procedure.

## Results

The mean, standard deviation, and analysis of covariance within the group were used to analyze the data. Table 1 shows a statistical comparison of SpO2 values in a single group. Significant differences between the pre-test and post-test means of the group in relation to SpO2 and the obtained p-value are less than 0.05 (p=0.0001).

**Table 1:** Description of single group in relation to SpO2

Group	Test SpO2	N	Mean	SD	Min	Max
Single Group	Pre	250	94.796	3.485	84	98
	Post	250	96.844	2.097	91	99

Table 2 demonstrates the analysis of covariance.

**Table 2:** Analysis of covariance of single group in relation to SpO2

Source	DF	Sum of Squares	Mean of Square	F Value	p-Value
Single group	1	1005.651	1005.651	2793.961	P<0.0001
Within group	248	89.265	0.360		
Total	249	1094.916			

**Figure 1:**
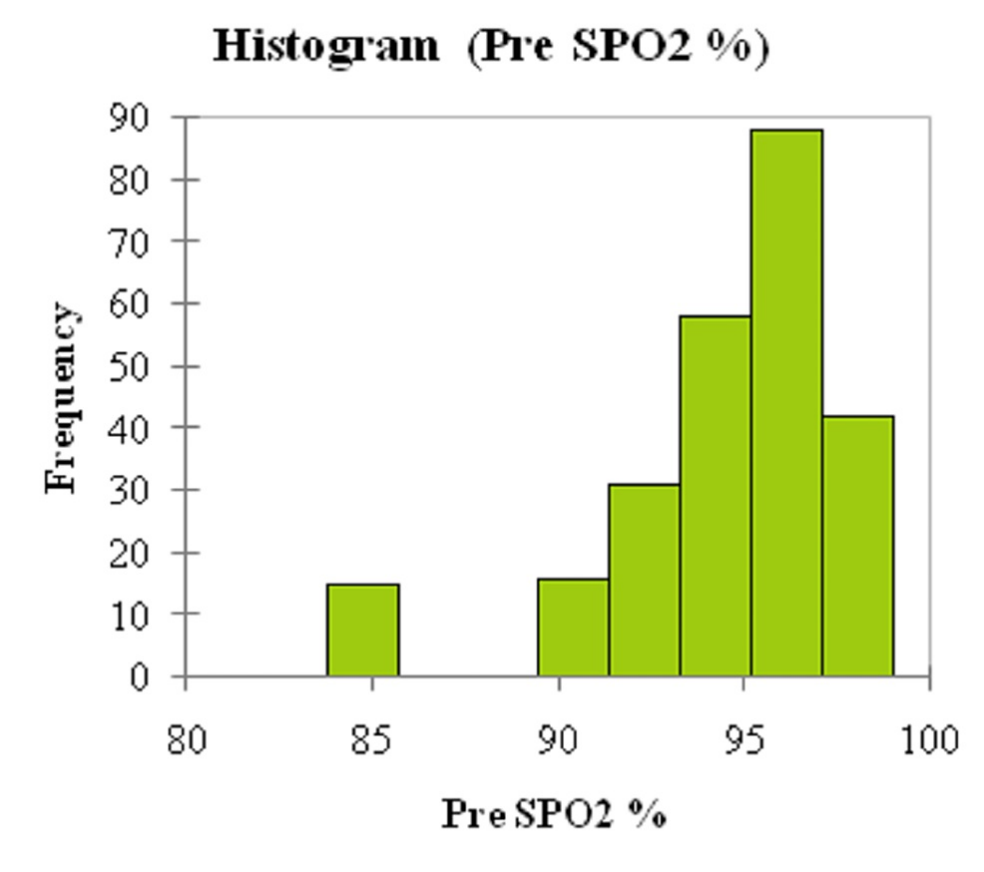
Graphical histogram for the pre-SpO2

**Figure 2:**
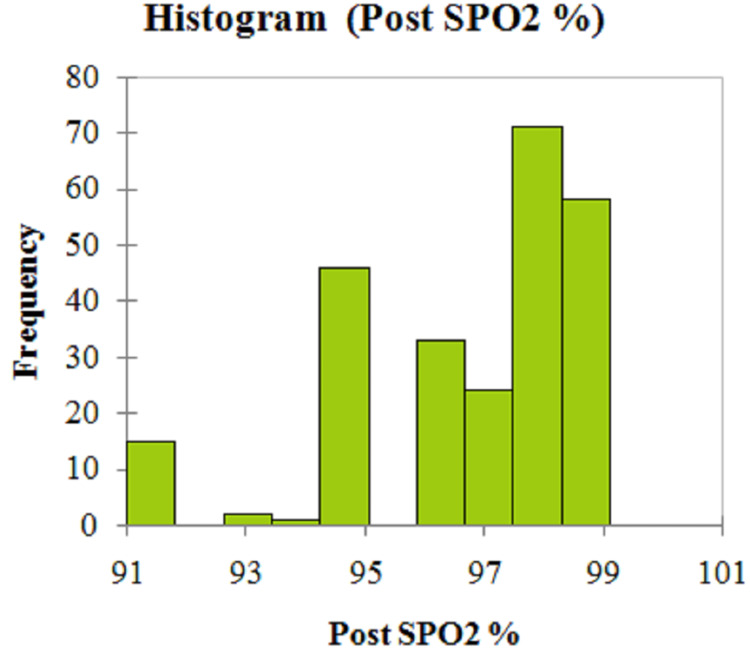
Graphical histogram for the post-SpO2

The graphical histogram presentation of pre-and post-SpO2 is illustrated in Figures 1, 2.

## Discussion

The study was performed at Sri Aurobindo Hospital Indore, consisting of a total of 250 COVID-positive patients based on the inclusion criteria. The results of the patients’ studies found that balloon-blowing exercise with conventional chest physiotherapy improved oxygenation in most of the patients. This can be due to increasing epigastric excursion and increased tone, leading to enhanced endurance and increased strength of the respiratory muscles, as after deep inspiration by the nose, when exhalation is done within the balloon, breathing is done against resistance, which further re-expands and keeps the airways and alveoli open by creating backflow pressure, thus increasing aeration and hence oxygen saturation [9,12,13]. The balloon-blowing exercise improves pulmonary functions and respiratory muscle strength, along with chest and lumbar mobility, and corrects the abnormal breathing pattern [9].

The diaphragm contracts more forcefully due to the resistance supplied by the balloon, which is active during the exhale phase. Both the inspiration and expiration phases include the continuous activity of the external and internal intercostal muscles. Respiratory exercise with an assistive device is a balloon further adds visual feedback, which also improves the patient's performance [14,15].

While performing the balloon-blowing exercise during inspiration, the diaphragm contracts concentrically, whereas the transversus abdominis contracts eccentrically. The muscle function is reversed during exhalation; the diaphragm contracts eccentrically, and the transversus abdominis contracts concentrically [16].

The patients in the current study were restless, had poor breathing and posture, and were unable to maintain the optimal zone of apposition, which is crucial because it is directed by the abdominal muscles, and when reduced, results in less inspiratory action of the diaphragm on the rib cage. The balloon-blowing exercise, in which slow exhalation is done, further relaxes the neuromuscular system and decreases resting muscle tone [8]. In the present study, the resistance of the balloon during exhalation leads to an increase in abdominal musculature activation and further increases the ability of abdominal muscles to oppose the diaphragm and maintain an optimal zone of apposition.

More transversus thoracis activation is required to reduce balloon resistance, which also aids in exhaling. Additionally, it aids in the lengthening and contraction of the intercostal muscles, which are used during both respiratory stages [17,18]. A previous study stated that balloon blowing exercise is a conservative exercise intended to help a patient achieve ideal posture and respiration, which supports the findings of the present study [19]. Additionally, Kripa et al. provided evidence for the idea that individuals with lower respiratory problems can benefit more from frequent balloon exercise than those with higher respiratory disorders [20].

Similarly, a study stated that the transverse and rectus abdominal muscles, during forced expiration contract actively. The diaphragm and intercostal muscles naturally relax during expiration, which is a passive action. The air blown into the balloon during the balloon-blowing exercise performed in the current study increased the rubber's elastic force and offered the abdominal muscles more resistance. According to the reports, as balloon capacity is increased, abdominal and expiratory muscles become more active. As a result, it's possible that the balloon-blowing exercise used in this study helped to strengthen the abdominal and expiratory muscles [11].

Blowing balloons exercises the intercostal muscles, which expand and lift the ribs and diaphragm. This makes it possible for the lungs to inhale oxygen and exhale carbon dioxide. Blowing up balloons efficiently increases the lungs' capacity to expand and take in air. Regular balloon exercises help activate the transverse abdominis and diaphragm. The diaphragm's main function is to draw air into the lungs. By pushing the diaphragm to its absolute maximum during balloon exercises, more air is drawn into the lungs [9].

Everyday balloon exercises help to improve the diaphragm's contractions and initiate a proper breathing cycle. Exercises with a balloon increase the diaphragm's effectiveness, which enhances the function of the lungs. Therefore, balloon-blowing exercise enhances oxygen saturation with appropriate lung function, resulting in a higher quality of life and quicker recovery. Exercise with balloons has many positive health effects, but it also helps with stress reduction and mental stability. Exercises with a balloon help to expand lung capacity, which has positive effects on the entire body and boosts confidence. This will aid in easing respiratory system pain and fatigue [9,19].

To improve ventilation and oxygenation, balloon blowing gradually expands lung capacity and strengthens the lungs' capacity to sustain a sufficient supply of oxygen. This enables the lungs to take in oxygen, change its chemical makeup while it is still in the lungs, and release carbon dioxide as soon as exhalation starts. By enabling the lungs to expand freely with enough oxygen to suit the body's needs, having sufficient lung capacity helps endurance [10]. Thus, by regularly exercising by blowing up balloons, the lungs can be worked out to build capacity exactly like muscles. To prevent respiratory issues after COVID-19, improve quality of life, promote early recovery, and shorten hospital stays, balloon-blowing activity can be advised. The study's limitations included the absence of a control group, the failure to compare balloon breathing to alternative breathing methods, and the use of solely oxygen saturation as an outcome metric.

## Conclusions

The study highlights that after performing balloon blowing exercises, a significant improvement in oxygen saturation in COVID-19 patients was observed. The balloon-blowing activity can benefit patients with COVID-19 and lessen the need for ICU treatment because it is simple to do, comfortable for everyone, and inexpensive. Additionally, this will relieve the strain on healthcare resources and personnel as the number of severely ill COVID-19 patients rapidly increases all over the world. Therefore, it may be concluded that physiotherapeutic balloon-blowing exercises are a useful way to enhance oxygen saturation in COVID-19 patients.
